# Supplementary material for: New Insights into Methyl Jasmonate Regulation of Triterpenoid Biosynthesis in Medicinal Fungal Species Sanghuangporus baumii (Pilát) L.W. Zhou & Y.C. Dai
Source: J Fungi (Basel). 2022 Aug 23;8(9):889. doi: 10.3390/jof8090889 (PMC9504552; doi:10.3390/jof8090889)
Supplement: Supplementary file 1 [file jof-08-00889-s001.zip › jof-1814825-supplementary.pdf]

Table S1. Primers used for this study.

Primer name	Sequence（5′→3′）	Description
HMGR-F	TGGCTTTGAACGCCGTCTTA	qRT-PCR reaction
HMGR-R	CTCTCCATTTCGTCGCTATCACC	
IDI-F	GCTGCTGCGGCGTTTGCTACTAT	qRT-PCR reaction
IDI-R	ATCTTCTCGGATGCTCGTTGCTG	
SQS-F	TCCTGCTGCTCATCACACATCCTC	qRT-PCR reaction
SQS-R	CCAATAAGTGCCAACATCGCTTCA	
SE-F	TAAGAACTCGGGGACTGAAACGAAG	qRT-PCR reaction
SE-R	CTTGAGAATCGCACCGACGAAGTAG	
LS-F	ACACAGTTCGCCCTTGAGAGCC	qRT-PCR reaction
LS-R	CATCTTCACGGCCCGTTCGATAGGT	
TRIT1-F	GGAAGGTCCTCCGTAATCTTGAAA	qRT-PCR reaction
TRIT1-R	AAGCGACTGTCCGCAGCCGTG	
GGPS-F	ATGGTAAATGATAAAACTGGTGGTC	qRT-PCR reaction
GGPS-R	TTGCTCTGAAGATTGAAATAGTCGT	
α-tubulin-F	CCAGCAAGCGTTACCGATT	qRT-PCR reaction
α-tubulin-R	TCCACGACGTCCATCGTTC	
HMGR-pro-F	GGTACTCATGTTTTGCTCGTGTTT	promoter isolation
HMGR-pro-R	GGATTGTAGGTGAAAGGAAAGTTG	
IDI-pro-F	ACTGATGGGCGAAAAAGGAAGCGAT	promoter isolation
IDI-pro-R	CAGACATGGTGAAGTCGCGCCG	
SE-pro-F	AGGTCGGTGGAGGTCATTGTGCTA	promoter isolation
SE-pro-R	CCGCCAACAATGAGAACGTCGTAA	
TRIT1-pro-F	TATTGCCCACCCTAAGTGAGAGAA	promoter isolation
TRIT1-pro-R	AAGAATGTCCAGTCCCCTATAGAC	
GGPS-pro-F	ACACGGACTTTATCGTAATCATCG	promoter isolation
GGPS-pro-R	ATCACTTGCTCATTTTCGGTCGTC	
pMD18-F	CGCCAGGGTTTTCCCAGTCACGAC	PCR verification
pMD18-R	AGCGGATAACAATTTCACACAGGA	

Table S2. Spearman correlations analysis of total triterpenoids content and five genes involved in triterpenoids biosynthesis.

Project	MeJA concentration	Gene transcript level				
		HMGR	*IDI*	*SQS*	*SE*	*LS*
MeJA concentration	1	-0.081	-0.466*	-0.728**	-0.361	-0.793**
Total triterpenoids content	0.132	0.910**	0.567**	0.143	0.543**	-0.365

Note: * means p<0.05, ** means p<0.01.

Table S3. Spearman correlations analysis of cZ and *TRIT1* gene.

Project	MeJA concentration	*TRIT1* gene transcript level
MeJA concentration	1	-0.914**
cZ content	-0.921**	0.956**

Note: ** means p<0.01

Table S4. Spearman correlations analysis of GAs and *GGPS* gene.

Project	MeJA concentration	*GGPS* gene transcript level
MeJA concentration	1	-0.900**
GAs content	-0.850**	0.813**

Note: ** means p<0.01.

Table S5. Spearman correlations analysis of mycelia growth rate, biomass and cZ, GAs.

Project	Mycelia growth rate	Biomass	cZ content	GAs content
MeJA concentration	-0.939**	-0.951**	-0.921**	-0.850**
Mycelia growth rate	1	0.990**	0.926**	0.822**
Biomass	0.990**	1	0.913**	0.810**

Note: ** means p<0.01.
